# Scalable metagenomic taxonomy classification using a reference genome database

**DOI:** 10.1093/bioinformatics/btt389

**Published:** 2013-07-04

**Authors:** Sasha K. Ames, David A. Hysom, Shea N. Gardner, G. Scott Lloyd, Maya B. Gokhale, Jonathan E. Allen

**Affiliations:** ^1^Center for Applied Scientific Computing, ^2^Lawrence Livermore National Laboratory and ^3^Global Security Directorate, P. O. Box 808, Livermore, CA 94551, USA

## Abstract

**Motivation:** Deep metagenomic sequencing of biological samples has the potential to recover otherwise difficult-to-detect microorganisms and accurately characterize biological samples with limited prior knowledge of sample contents. Existing metagenomic taxonomic classification algorithms, however, do not scale well to analyze large metagenomic datasets, and balancing classification accuracy with computational efficiency presents a fundamental challenge.

**Results:** A method is presented to shift computational costs to an off-line computation by creating a taxonomy/genome index that supports scalable metagenomic classification. Scalable performance is demonstrated on real and simulated data to show accurate classification in the presence of novel organisms on samples that include viruses, prokaryotes, fungi and protists. Taxonomic classification of the previously published 150 giga-base Tyrolean Iceman dataset was found to take <20 h on a single node 40 core large memory machine and provide new insights on the metagenomic contents of the sample.

**Availability:** Software was implemented in C++ and is freely available at http://sourceforge.net/projects/lmat

**Contact:** allen99@llnl.gov

**Supplementary information:** Supplementary data are available at *Bioinformatics* online.

## 1 INTRODUCTION

Metagenomics is a powerful tool for assessing the functional and taxonomic contents in biological samples. Early shotgun metagenomics projects used giga-bases of genetic data (Venter *et al.*, 2004) to demonstrate accurate sample surveys with less bias than previous methods. The potential to detect even lower abundance organisms and provide more accurate surveys across a broad spectrum of biological environments is being advanced now by sequencers reported to generate up to 1.3 mega-bases per second (Knight *et al.*, 2012) (Calculated by dividing total base output by total number of sequencer hours run for the HiSeq 2500 rapid-run mode. Excludes library and sample preparation time).

Increased sequencing throughput presents a major scaling challenge to existing shotgun metagenomic classification algorithms (Drge and McHardy, 2012). The ability for an algorithm to scale can be measured by the difference between sample classification run time and sequencer run time and assumes sufficient computing resources for each sequencer run. Scaling is being addressed through the use of larger compute clusters, which can be managed by a third party service (Cloud computing) (Schatz *et al.*, 2010). As sequencer use grows, however, algorithms that run on a single node and scale with sequencer output could be paired with individual sequencers and eliminate the need for high bandwidth network connections, which are not always available.

In this article, we attempt to meet the scaling goal, running fast and accurate taxonomic sample classification on a single compute node to match analysis throughput with sequencer output. Two major design choices were made, which present possible limitations: (i) a larger than typically used single address space memory resource is exploited (0.5–1 terabytes) and (ii) larger search seeds are used than default sensitive BLAST settings for matching reads to a reference database. Relaxing conventional memory constraints allows a reference genome database to be annotated with taxonomic information and indexed to support fast metagenomic taxonomy classification of every sequencer read for all microbial taxa, including virus, prokaryotes, fungi and protists. Decreasing memory costs make this approach accessible to many practitioners because the cost of a single large memory compute node remains a fraction of the initial sequencer cost and need not require specialized system administration expertise. Large search seed sizes can potentially limit the ability to detect novel organisms but nonetheless is proving to be more effective, as the number of microbes with representative reference genomes grows for environments like the human microbiome (Martin *et al.*, 2012).

Our goal is to efficiently assign taxonomic labels to the reads down to the species level for reads with reference representation and maintain accuracy in the presence of novel organisms by avoiding overly specific (e.g. species and strain) taxonomic assignments. This alleviates the computational bottleneck by limiting the number of unlabeled reads subjected to additional computational interrogation. The results show comparable or better accuracy than existing methods, and even with novel genomes in a sample, accurate and scalable classification is obtained in the vast majority of cases. The methods are made available as an open source software package, Livermore Metagenomics Analysis Toolkit (LMAT).

Existing bioinformatic approaches address scalability in three ways: query size reduction, reference database size reduction and faster database search. Query size reduction is achieved with metagenomic assembly and clustering, which merges overlapping and redundant reads into longer contiguous genomic segments (Pell *et al.*, 2012). Metagenomic assembly improves the strength of the taxonomic signal contained in individual short reads but careful parameter settings are required to avoid mis-assembly, and assembly costs could remain high (Mende *et al.*, 2012; Teeling and Glöckner, 2012). Reference database size reduction is achieved through the use of genetic markers storing only the more informative sequences (Berendzen *et al.*, 2012; Liu *et al.*, 2011; Segata *et al.*, 2012). Marker-based approaches offer efficient summarization of metagenomic contents, but only cover a portion of the query set, leaving novel and other informative reads buried within the larger pool of unclassified reads, which could require additional examination (Mohammed *et al.*, 2011). A less lossy approach reduces sequence redundancy by storing only the genetic differences among reference genomes. This approach was shown to speed up BLAST and BLAT genome database searches (Loh *et al.*, 2012). Faster database search methods apply larger search seeds, and examples include BLAT (Sharma *et al.*, 2012), BWA (Davenport *et al.*, 2012) and other read mapping tools (Martin *et al.*, 2012), but analyzing the search results remains a challenge with some approaches selecting the lowest common ancestor (LCA) of multiple matches and others using variants of a best match selection procedure to improve rank specificity of the reported taxonomic label. Moreover, parameter settings of the search tools can dramatically alter the outcome of the reported label and must be considered carefully (Mande *et al.*, 2012).

Our approach uses faster search using larger seeds (k-mers) with a non-redundant search of taxonomic identifiers associated with the k-mers found in the reference genome database. Our k-mer/taxonomy database supports efficient retrieval of detailed taxonomic information and allows for an exhaustive comparison between competing taxonomic assignments using a novel rank-flexible classification procedure. Our new classification algorithm invokes variants of LCA and best match selection depending on the context of the search results. The approach differs from compositional binning methods (Leung *et al.*, 2011), as it uses larger values for k (17–20) and, unlike alignment search, each k-mer is mapped to the individual source genomes minus the genome position. The method compensates for the lack of positional information by resolving the multiple k-mer/taxonomy associations recovered during search to assign each read the most rank-specific taxonomy identifier possible.

## 2 MATERIALS AND METHODS

### 2.1 k-mer/taxonomy database

A reference genome database consists of a collection of genome sequences with each genome sequence assigned a taxonomic identifier. The first step is to convert this ‘raw’ reference genome database into a searchable k-mer/taxonomy database by storing every overlapping k-mer along with select taxonomic IDs. A reference database was constructed from complete and partial microbial genome sequences from the NCBI genome database on October 17, 2011. The ‘raw’ genome database included 301 935 distinct genomic segments (plasmids, chromosomes and other genomic segments) and contains 67 073 viral, 4366 bacterial and 236 archaea segments. The remaining segments are shared among draft eukaryotic microbial genomes that were included as assemblies with many contigs and supplementary mitochondrial genomes from eukaryotes. The reference set includes 1272 bacterial species, 121 archaeal species, 3048 viral species and 335 eukaryotic species. Microbial genome segments range in length from a small number of single read contigs of length less than 100 bases up to a 13 033 779-base chromosome (for *Sorangium cellulosum*).

**Fig. 1.**
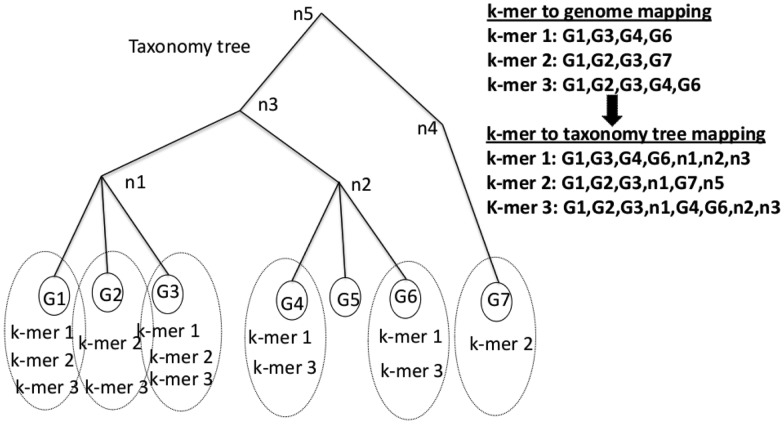
Example k-mer/taxonomy database. Input includes the taxonomy tree with interior tree nodes 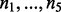, and leaf node genomes 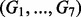, all of which are labeled with taxonomy IDs. k-mers (k-mer1, k-mer2, k-mer3) are linked to their source genomes (dotted circles) and their taxonomy hierarchy up to the LCA

**Fig. 2.**
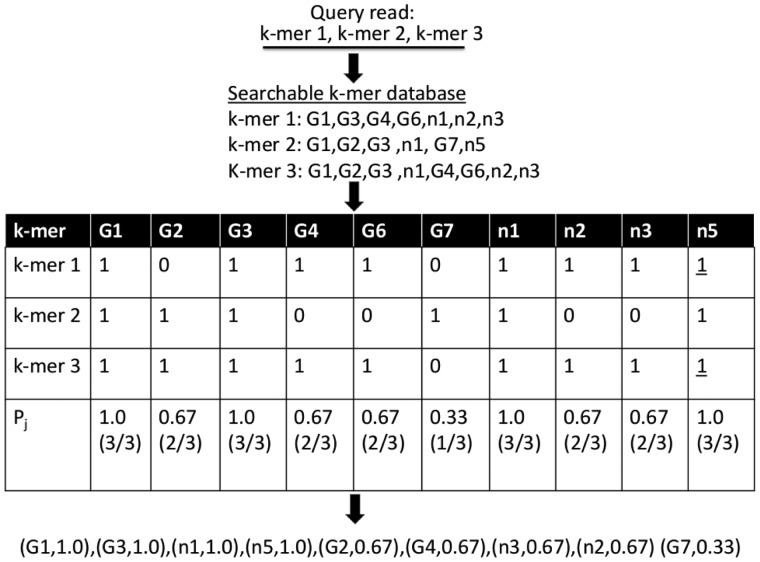
Example scoring procedure. The query read is converted to k-mers (k-mer1, k-mer2, k-mer3), and their associated taxonomy information retrieved from the database. A classification table is created with columns for candidate taxonomic IDs and rows representing a specific k-mer, with a binary entry reporting the presence or absence of the k-mer in some genome associated with the taxonomic node. The last row shows k-mer row sum divided by the total number of k-mer rows. The underlined entries highlight nodes that are created at run time

Taxonomic IDs represent nodes in the taxonomy tree and cover all ranks from an individual genome or strain up to the highest order domains. Figure 1 shows an example representation for the searchable database. As input, database construction requires (i) an NCBI taxonomy tree, (ii) a reference genome sequence database and (iii) mappings between the genome sequence identifiers and taxonomy identifiers from the taxonomy tree. Then, all overlapping k-mers from the genome database are computed, and the LCA for the taxonomic IDs for each k-mer is identified. Finally, a post-order tree traversal up to the LCA counts the number of genomes that contain the k-mer for each taxonomy node in the traversal. Our initial expectation was to use k-mer counts to weigh each k-mer’s contribution to a candidate taxonomic label assignment. However, the weighting procedure was found to be sensitive to genome representation bias and therefore a binary scoring scheme was chosen (see Fig. 2).

In our experiments, we use a ‘full’ k-mer/taxonomy database (kFull) and a smaller database built from a ‘marker library’ (kML). A marker library contains the most taxonomically informative set of k-mers present in the raw genome database. The marker library is created by separating k-mers into disjoint groups. A k-mer is in exactly one group. Each group has a unique label, which consists of the names of all genomes (or more generally, sequences including genome, plasmid, segment or chromosome) that contain the k-mers in the group. For example, k-mers that occur in exactly the genomes A, B and C are in one group ‘A, B, C’, k-mers that occur only in genomes B and C are another group ‘B, C’ and k-mers in genomes A, C and D are a third group ‘A, C, D’. As a more concrete example, suppose k-mer1 is present in *Yersinia pestis* KIM, *Y.pestis* CO92 and *Yersinia pseudotuberculosis* IP32953. k-mer2 is present in *Y.pestis* CO92 and *Y.pseudotuberculosis* IP32953. Both k-mers have the LCA of *Yersinia*, but in building the marker library, they are in two separate groups based on the exact set of genomes that contain the k-mer: k-mer1’s group is labeled ‘*Y.pestis* KIM, *Y.pestis* CO92, *Y.pseudotuberculosis* IP32953’, and k-mer2’s group is labeled ‘*Y.pestis* CO92, *Y.pseudotuberculosis* IP32953’. All the k-mers in groups containing more than 1000 k-mers are included in the marker library. So, if there are 1000 k-mers in k-mer1’s group, then all those k-mers go into the marker library. If there are 999 k-mers in k-mer2’s group, then none of those k-mers go into the marker library. k-mers whose LCA is above the taxonomic rank of family are not included in the marker library. A k-mer/taxonomy database is created from the marker library’s set of k-mers.

### 2.2 Scoring a read’s taxonomic IDs

In the k-mer/taxonomy database, each k-mer of length *k* is associated with a list of taxonomic IDs as outlined in Section 2.1. The first step of determining a taxonomy ID for a read from the query set is to assign a score to each taxonomy ID of each k-mer in the read. The score is derived from the proportion of k-mers of the read that occurs under that taxonomy node normalized by the proportion of k-mers of a random read that also appears under that taxonomy node.

To illustrate the details of the scoring algorithm, the example in Figure 2 shows a query read with three k-mers. The tax IDs of the read’s constituent k-mers are retrieved from the k-mer/taxonomy database. For each read *s* of length *l*, a binary classification table *C* of size 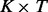 is constructed, in which the *K* rows represent the k-mers retrieved from the read and the *T* columns represent candidate taxonomic IDs (k-mers are stored in the database in canonical order removing strand specificity. Only canonically ordered k-mers are used to query the database.). A ‘1’ entry indicates the tax ID (column) belongs to the associated k-mer (row). For each tax ID *j*, the proportion of k-mers having that tax ID is computed (shown in Fig. 2 in the last row): 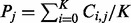. In the example, 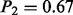 for genome G2.

The scoring method uses a random model to limit genome representation bias in the database and avoid assigning taxonomic labels by random chance when a novel organism is not represented in the reference database. The score *S_j_* of read *s* for a taxonomic ID *j* is defined as 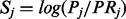, where *PR_j_* represents the proportion of k-mers associated with taxonomy ID *j* in the random model. The random model estimates *PR_j_*, the chance of assigning a read of length *l* to taxonomy ID *j* owing to random chance (for simplicity the random score is not shown in Fig. 2). The random model is precomputed for read length *l* and assumes a random nucleotide composition, which can optionally sample explicitly from a range of GC content values. Reads are randomly generated, and then are searched against the database to calculate a *P_j_* value for each random read *r* and observed taxonomy ID *j*. *PR_j_* is set to the maximum observed value 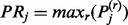. Further details of random model construction are included in the Supplementary Material.

### 2.3 Rank-flexible read classification

**Fig. 3.**
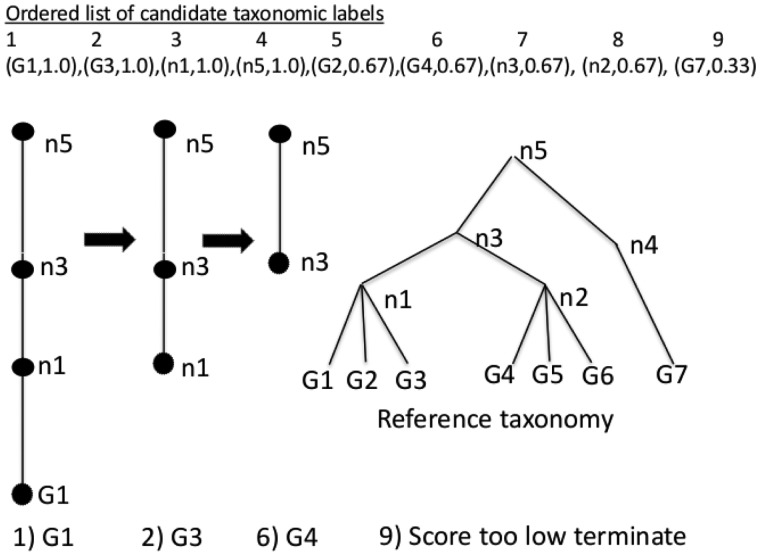
Label selection process. The list of candidate taxonomic labels ordered 1 through 9 is sorted by label score (G1,1.0), … ,(G7,0.33). Steps 1, 2, 6 and 9 where an action is performed are shown. At step 1, the taxonomy lineage is constructed from the best first label G1. Step 2, G3 conflicts with G1 and the lineage is pruned to n1. Step 6, G4 conflicts with n1 and the lineage is pruned to n3. For demonstration at step 9, score 0.33 for the G7 label is below threshold and the procedure terminates and returns n3 as the classification

Taxonomy classification combines LCA selection with the read label score evaluation. Candidate labels are examined in order by decreasing read label score as shown in Figure 3 (for illustration, the numerator of the read label score *P_j_* is shown). The most specific taxonomic label is selected such that no other taxonomic label from a conflicting lineage has a comparable read label score. Comparable is defined as a score within one standard deviation of the best candidate using the read label score distribution for the read. The best candidate is found using the taxonomic lineage from the highest scoring taxonomy label. The path from the highest scoring node to the LCA is created (LCAs for individual k-mers identified off-line are used as a starting point to find the LCA for all retrieved k-mers online, which in some cases can reduce run time costs) and each subsequent label is evaluated for consistency with the lineage. When conflicting labels are encountered, the lineage is pruned further up the tree to resolve the conflict. In the example shown in Figure 3, the lineage from *G*_1_ to the LCA *n*_5_ is constructed first. When *G*_3_ is examined, it is identified as conflicting with *G*_1_ and the candidate lineage is pruned to node *n*_1_. The process continues until the ninth label is encountered (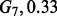), which for the purpose of illustration has a score below the threshold of comparable scores. The classification procedure terminates with the read assigned label *n*_3_.

Classifications are divided into categories. The ‘LCA Match’ category means the LCA has a lower read label score but there are multiple conflicting labels with higher comparable read label score. This is the traditional LCA assignment algorithm (Huson *et al.*, 2007) where multiple significant matches from competing lineages are found. The ‘Direct Match’ category means the classification reflects matches to the taxonomy without a conflicting lineage but the classification could be from any rank, including strain, species, genus or higher. Higher order rank assignments in the Direct Match category imply that the read label score for lower rank assignments were below the comparable score threshold and suggest sequence novelty in the classified sequence. ‘Novel Match’ is reported when multiple child nodes from competing lineages have comparable read label scores above the threshold but the LCA has a higher read label score. This indicates novelty in the query read and could occur when a substantial subset of the read’s k-mers are found in competing lineages, but the combination of k-mers observed at the LCA node is more significant.

### 2.4 Database ingest

A key feature of our approach is to store the k-mer/taxonomy database in a file rather than build it anew for each query run; this permits shifting computational costs to the off-line taxonomy/genome ingest phase. The database is created once, and then is used repeatedly during classification. To enable database search during classification, the file is mapped into the memory address space of the classification program, allowing k-mers and associated taxonomy IDs to be accessed directly. Previous work at Lawrence Livermore National Laboratory (LLNL) has modified the Jemalloc memory management library (Evans, 2006) to enable memory allocation from an address range memory-mapped to a file residing on a storage device. Jemalloc is a drop-in replacement for regular malloc routines for allocating memory. Our modification to Jemalloc allows for an additional step to specify the database filename (Jemalloc memory-maps to temporary files). We have chosen to use the memory-map file approach rather than implementing an out-of-core indexing algorithm because of the ease of programming using the memory-map abstraction for the persistent storage of data structures. We place the memory-mapped files onto a ramdisk for in-memory performance. An ingest utility creates the database, which includes a hash table whose keys are k-mers and values are sets of taxonomy identifiers.

Supplementary Table S2 shows numbers of k-mers present and the total storage required for several of the databases. The current default settings use 619 GB (kFull) and 39 GB (kML). The databases use 6 bytes per taxon, including genome counts information. We found that these counts are extraneous information and only 2 bytes per taxon identifier are required. Using 2 bytes per taxon allows for 65 566 distinct taxa (the current database has 18 498 distinct taxa). Future work is expected to reduce the database size to 413 GB (kFull) and 26 GB (kML). The ingest pipeline for the full database took ∼17 h using up to 256 2.3 GHz Advanced Micro Devices (AMD) compute nodes each with 32 GB running single-threaded tasks that dumped intermediate results to files in the parallel file system.

### 2.5 Test data

To compare performance with existing state-of-the-art published tools, accuracy was compared with PhymmBL (Brady and Salzberg, 2011), MetaPhlAn (Segata *et al.*, 2012) and Genometa (Davenport *et al.*, 2012). PhymmBL balances classifying known species with classifying novel organisms but uses BLAST, which does not scale well with sequencer output (Angiuoli *et al.*, 2011). MetaPhlAn uses a small marker library making it scalable but it does not attempt to label every read. It is also optimized to do relative abundance estimation, which LMAT currently does not do. Genometa replaces BLAST with a potentially faster search algorithm (Bowtie2 or BWA) and attempts to assign a taxonomic label to every read. Ideally, the same reference database would be used for all programs. However, adapting our database to work with Genometa and PhymmBL required significant customization and was not technically feasible with MetaPhlAn. Therefore, the existing reference database of each tool was used. The Genometa database is the oldest database created in 2010, our reference database was created in fall 2011, and PhymmBL and MetaPhlAn use databases created in mid 2012. The published PhymmBL dataset that uses a read length of 100 was chosen as the test query dataset. As the test data were created before each reference database was created, the query species should be present in each reference database.

Three additional simulated test query sets were used to evaluate the accuracy of our method in the presence of query sequences absent from the reference database for viruses, prokaryotes and eukaryotes (fungi and protists). MetaSim was used with a 100 bp (Barthelson *et al.*, 2011) and 80 bp (Richter *et al.*, 2008) Illumina error model to generate the novel bacterial and viral dataset, respectively. Eukaryotes were taken from single species sequencing data deposited in the Short Read Archive (SRA) and include *Trypanosoma evansi*, *Candida albicans*, *Coccidioides immitis*, *Aspergillus fumigatus* and *Entamoeba histolytica* (read lengths ranged from 36 to 200). For the bacterial dataset, 100 sequences not found in the reference database (determined by GenBank Identifier and header comparison) were selected at random to serve as the candidate test set with 1 000 000 simulated reads and equal concentrations of the 100 bacteria. The 100 strains were made up of 75 distinct species, 14 of which were species not found in our reference database. For the viral case, 6921 reads were generated, assuming equal concentrations of 25 viral genomes, which made up 25 distinct species, 10 of which were not found in the reference database. Not every test sequence in the ‘novel’ datasets proved to be divergent from the reference database. A detailed description of the test data is given in the Supplementary Material. To measure run time, three non-synthetic metagenomic samples representing a viral metagenome (SRX022172), a human microbiome metagenome (ERR011121) and a single species raw read ‘metagenome’ (DRR000184) were taken from the SRA.

## 3 RESULTS

### 3.1 Classification accuracy

Read accuracy is reported by counting the number of reads correctly assigned to a taxonomic label consistent with its true origin. For example, a read originating from *Bacillus anthracis*, but assigned to *Bacillus* would be counted as correct at the genus rank. Read accuracy is tracked for each taxonomy rank to compare accuracy with rank specificity. Sample accuracy is reported for true-positive and false-positive counts on species calls. This introduces one free parameter, which is not automatically selected—minimum number of reads needed to make a species call (or minimum species abundance for MetaPhlAn). Results for all programs are reported as performance curves to identify the trade-offs of reducing the false-positive count with increased thresholds while potentially reducing the true-positive count.

**Fig. 4.**
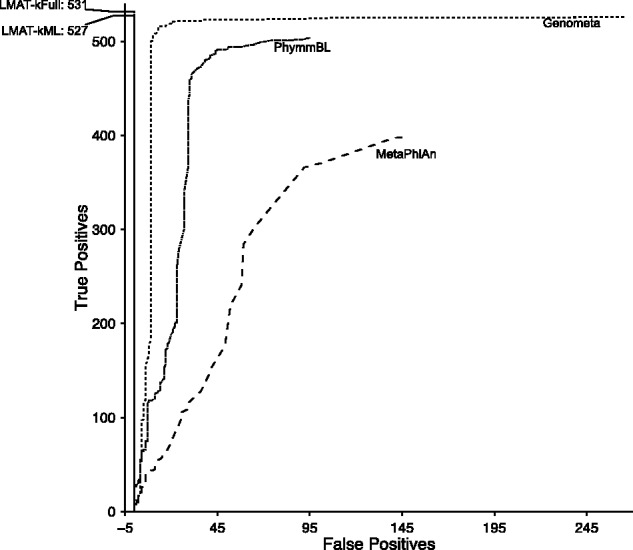
Species-level accuracy comparing reference databases/algorithms’ performance on PhymmBL query set. Classifier performance is shown using the full database (LMAT-kFull), and a marker database (LMAT-kML), and is compared with other software, Genometa, PhymmBL and MetaPhlAn. LMAT-kFULL performance is underneath the LMAT-kML plot, highlighting similar performance

Figure 4 shows the true-positive/false-positive performance curve for different minimum read thresholds compared with the other contemporary methods and our own reduced size marker library (kML) and full-sized library (kFull). Values for k = 20 (kFull) and k = 18 (kML) were used as default values (accuracy comparisons for different values for k are shown in Supplementary Figure S1). LMAT-kFull and LMAT-kML showed near identical accuracy, with the full-sized database correctly detecting a small number of additional species. Genometa’s advantage over PhymmBL could be explained by its stricter read mapping criteria, which favors detection of known genomes. Although we were able to recreate higher performance runs for MetaPhlAn on their previously published test sets, MetaPhlAn’s lower performance on the PhymmBL test suggests that it may be more difficult to do both taxonomic identification and relative abundance estimation when microbial content consists exclusively of exceptionally low per genome coverage. (The PhymmBL test set contains just 50 reads per genome and includes multiple closely related strains.)

Table 1 shows the percentage of PhymmBL input set reads correctly labeled for select ranks and reads that were incorrectly labeled or failed to be assigned a label. The table shows that the majority of reads labeled by LMAT using the kFull database are identified by species (74.2% for the full database) with high accuracy (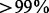). When LMAT used our marker library (LMAT-kML), fewer reads (40%) were classified but the assignments were highly accurate (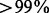). Although PhymmBL can potentially make rank flexible selections using the confidence scores assigned to different rank levels, we were not able to implement an automated threshold that demonstrated good results. Therefore, we report observed species accuracy calls as well as PhymmBL’s published results, which are slightly higher. Genometa’s accuracy was lower than other methods with fewer reads assigned species labels. This is likely because of its use of a best hit approach instead of rank-flexible selection. (MetaPhlAn does not report read-specific labels, thus it is excluded from the table.) Our method using the full database could not assign species-specific labels to 25.8% of the reads; 8.5% of the reads were assigned labels with ranks at the genus, family and order levels; 6.9% and 3.1% of the reads not listed in Table 1 were correctly assigned labels at other ranks (e.g. kingdom, sub-species, class etc.) for the kFull and kML databases, respectively. The high overall LMAT accuracy is explained by the rank-flexible selection, where low rank assignments are only made in the absence of conflicting evidence, with the read label score filtering out an additional 10% of the reads, thus leaving just 0.3% of the reads with an incorrect assignment.

**Table 1.** Per read accuracy for known bacteria shown in percentages

Application	Database	Species	Genus	Family	Order	Wrong	No label	No hits
LMAT	kFull	74.2 (99.7)	6.7 (99.9)	1.4 (100)	0.4 (100)	0.3	10.1	0
LMAT	kML	40.4 (99.8)	4.1 (99.9)	0.8 (100)	0.1 (100)	0.2	51.3	17.7
PhymmBL	Published	– (95.4)	–	–	–	4.6	–	–
PhymmBL	GenBank	88.3 (92.5)	–	–	–	7.5	11.7	–
Genometa	Published	66.7 (92.2)	–	–	–	7.8	33.3	–

*Note*: Per rank accuracy shows two values—percentage of all reads correctly labeled by rank (species, genus, family or order) or incorrectly labeled (Wrong) or failed to be assigned a label (No label and No hits). In parentheses shows percentage of reads assigned a label at the specified rank that were correct. No label = reads with no taxonomically informative label assigned and includes No hits, No hits = reads with no k-mer matches to the database. – = entry not applicable.

**Fig. 5.**
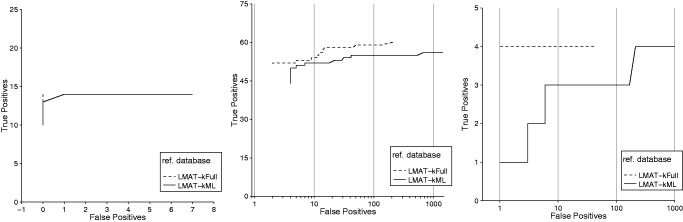
Classification accuracy when novel genomes are included in the input sets. The two database types are considered Full (kFull) and the marker library (kML). Left panel shows performance for simulated viral metagenomes with 25 total species and 10 novel genomes. Middle panel shows 75 total species including 14 novel genomes and right panel shows 5 protist/fungi with one 1 novel genome included. *x*-axis counts the number of species reported that are not present (False Positives) and the *y*-axis counts the number of true species present that are reported (True Positives). The performance curve reflects 300 different threshold values for minimum number of labeled reads required to make a species call

One key challenge is to maintain accuracy in the presence of novel genomes by preventing overly specific rank calls. Because it proved to be challenging to manipulate the other publicly available software tools to use our reference database particularly in the case of the viral and eukaryote test sets, it was not possible to directly compare results. Instead, we evaluate our two databases—kFull and kML. Figure 5 shows the species sample accuracy curve for our method on the three datasets (virus, bacteria and eukaryotes), which include novel organisms. The number of test species that are also represented in our reference database were 15, 61 and 4 for the viruses, prokaryotes and eukaryotes, respectively, and indicate the practical upper bound on the number of correctly identifiable species. For the novel viral case, approximately 1× coverage was used for each viral genome. With this relatively good coverage level, although only 14 of the 15 known viral species were correctly called, no false species calls were made. For the bacteria case, 0.35× coverage was used, and the eukaryote case used 470 779 reads chosen at random to simulate low coverage.

In eukaryote and prokaryote cases, the false-positive count was extremely low but not perfect. In the eukaryote case, the *T.evansi* not in our reference database was classified *Trypanosoma brucei* and it appears that significant portions of these two genomes are highly similar, making the distinction difficult. The kML eukaryotic results are expected to improve as more microbial eukaryotic genomes are sequenced to allow for better targeting of markers that discriminate between near neighbors. For bacteria, there were two false-positive species calls, *Mycobacterium abscessus* and *Rahnella sp.* Y9602, which share significant portions of their genomes with the novel species coming from the same genus (*Ranunculus aquatilis*, *Mycobacterium chubuense* and *Mycobacterium massiliense*). When the species accuracy dropped, the genus calls remained highly accurate (∼99% for eukaryotes and prokaryotes). For these cases, we hypothesize that future work using relative counts of genus and species calls compared with no call counts could automatically differentiate the presence of a novel species from a known species match. Supplementary Table S1 shows high accuracy at the individual read level.

### 3.2 Speed performance

**Fig. 6.**
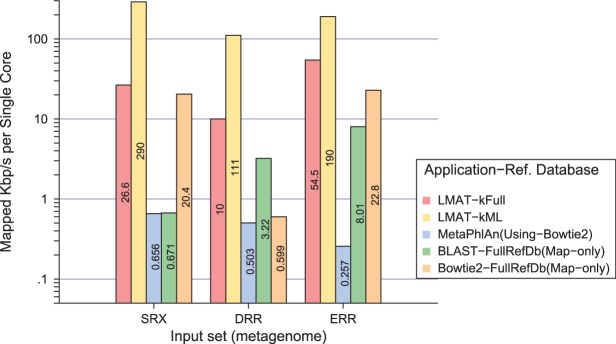
Run time performance. Tests run on three real metagenomic datasets SRX, DRR and ERR. Run times are shown for the metagenomic classifiers (LMAT-kFull, LMAT-kML and MetaPhlAn using Bowtie2 for read mapping and its reference database) and simple sequence searches for Bowtie2 and blastn (BLAST) using the same full reference genomes found in kFull. We report run time normalized to the percentage of mapped or labeled reads. Note log scale on *y*-axis; values given within each bar

Run time of our classifier is compared with MetaPhlAn as an existing scalable metagenomic classifier using the marker library approach. Additional comparisons were made with the search tool Bowtie2 (Langmead *et al.*, 2009) and blastn. It is noteworthy that these mapping tools (Bowtie2 and BLAST in Fig. 6) do not perform classification, and tools such as PhymmBL and Genometa would have to be run on the mapped reads, which might increase total run time (considering appropriately balancing run time and accuracy trade-offs). Nonetheless, if our methods are comparable in speed with raw search times, they should present a distinct advantage. Measurements were conducted on a single node large memory machine, a quad-CPU Intel Westmere (10 core per CPU) with 1 TB of DRAM, running linux kernel 2.6.32.

Figure 6 shows efficiency measured by run time normalized by the percentage of reads that are mapped to the database. Supplementary Section 3 shows the raw run times and percentages of matched or labeled reads for each search tool. Using the mapped rate helped identify cases where a method’s speed could partly be explained by labeling fewer reads. Using the mapped or labeled rate efficiency metric, the results show that LMAT speed compares favorably on all datasets to the other tools. The closest performance to LMAT with the kFull database for mapped or labeled reads is Bowtie2 using the SRX dataset, in which LMAT was 30% faster. Across all three sets, LMAT performs on average 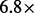 faster than Bowtie2 and 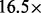 faster than BLAST. Using the kML database and normalized for the percentage of reads mapped, LMAT performs on average 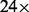 faster than Bowtie2 and 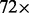 faster than BLAST. The BLAST results shown in Figure 6 use a search seed size of 28 (the current default setting) and are thus tuned for speed. PhymmBL version 3.2 uses a more sensitive search seed size of 11, which is more computationally costly. PhymmBL jobs did not complete within 48 h on the smallest timing dataset (using 80 threads) underscoring our expectation that BLAST-based approaches with the smaller search seed size do not scale for this problem.

Most surprising were the gut metagenome (ERR) observations, where the full database run with LMAT was faster than MetaPhlAn, despite the fact that the MetaPhlAn reference database is a reduced set of bacterial genes. This may be explained by a larger percentage of the k-mers mapping to fewer candidate taxonomic labels, as this is the primary run time cost associated with LMAT classification and could provide a speedup advantage over even fast alignment-based searches using Bowtie2. The other primary driver of performance is reflected by the difference in raw run time versus fractions of reads labeled or mapped.

Although we observe raw search output from Bowtie2 runs at a slightly higher rate for the ERR sample (see Supplementary Section 3), it only mapped 34.3% of the reads in contrast with the LMAT classifier, which assigns a label to 85.6% of the reads in the sample. For the Bowtie2 parameter settings, both ‘sensitive-local’ and ‘very-sensitive-local’ were considered and the faster time was reported. A key drawback of this parameter setting is it reports only the top three hits, which boosts speed but could result in overly specific species calls. Although LMAT run time depends on the sample, the observed performance rate was close to the target rate of 1.3 Mbp/s (1.34 Mbp/s on average). Thus, LMAT’s classification rate scaled well with sequencer output.

### 3.3 Application to a large metagenome

To confirm LMAT’s ability to analyze large (real) metagenomes and provide new biological insight, we downloaded the Tyrolean Iceman sequence data (Keller *et al.*, 2012) from the SRA, which constituted 150 giga-bases of raw genomic data. While 78% of the sequenced reads were reported to be human, only a small percentage (0.84%) of the reads was reported to originate from bacteria based on a sample of 8 million reads. Our hypothesis was that LMAT could examine all the reads on a single large memory compute node and efficiently provide a more complete analysis of the microbial contents. For this application, the human genome (v19) was added to LMAT’s database to classify human and microbial reads simultaneously. The analysis on the raw 150 giga-base dataset (2.3 billion reads) ran in <20 h on our single node large memory computer (see Supplementary Material for additional details). LMAT output agreed with the published finding that the vast majority of bacteria were from the phylum *Firmicutes* and under the class of *Clostridia*. Similarly, only a small fraction of reads were reported to be from the *Spirochaetes* phylum. LMAT results did not show evidence for the presence for non-phage, non-retroviral viruses, fungi or protists after adjusting for previously unidentified human contamination in draft eukaryote genomes present in the LMAT reference database. The key observed difference was in the *Borrelia* species previously reported to be the first documented case of Lyme disease in humans. Although LMAT’s findings support the presence of the *Borrelia* genus with 16 180 reads assigned a read label score greater than 0, a more complex relationship is shown between the new *Borrelia* sequence and previously sequenced *Borrelia* genomes. Although *Borrelia burgdorferi* was previously reported to be the likely species present, LMAT shows that among the reads assigned to the *Borrelia* genus, the majority of the reads are assigned to non–species-specific genomic regions with species-specific reads assigned to several *Borrelia* species, including *B.burgdorferi*, *Borrelia garinii* and others. The *Borellia* reads were compared against all sequenced Borrelia genomes to compute an SNP-based genetic distance matrix. The phylogenetic tree given in Supplementary Figure S10 supports LMAT’s finding that the *Borrelia* variant is divergent from *B.burgdorferi*.

## 4 DISCUSSION

LMAT leverages large single address space memory to efficiently and accurately assign taxonomic labels to individual reads in large metagenomic datasets even in the presence of novel organisms. Although the classification method is highly automated, attention to three parameters should be highlighted: minimum read label score, minimum difference between the best selected read label score and the competing alternatives and maximum number of taxonomic labels retrieved per k-mer. Although the first two parameter settings were preset early in the development stage, application to the Tyrolean Iceman dataset required revisiting these parameters. The data featured especially short reads (as short as 25 nt), degraded DNA, likely leading to lower quality scores and higher error rates and substantial human ‘contamination’ with respect to analyzing the microbial contents. As a result, the default read label threshold needed to be lowered from 1 to 0 to avoid ignoring a larger fraction of the reads. Interestingly, 25 nt length reads classified as human were assigned a read label score of 0 but could still be classified as human when no competing alternatives were found. However, if the sample came from a truly unknown source, the 25 nt reads with read label scores near 0 would require additional validation. The minimum difference threshold also needed to be increased when a large percentage of reads were initially classified as *Toxoplasma gondii*. On further examination, the matches identified human contamination in the genomes from LMAT’s reference database. Ideally, these reads would be classified as ‘superkingdom Eukaryota’ on the first pass (identified as both human and *T.gondii*). The random model, however, led to a slightly lower score for the human label compared with equivalent *T.gondii* label. Thus, while default parameter settings should be acceptable for many analysis cases, there may be conditions where awareness of these parameter settings is needed. The final user-defined parameter setting limits the number of candidate taxonomy identifier considered and was set to 50 early on to ensure efficient run times with the understanding that accuracy costs are incurred for genetic fragments associated with a complex taxonomic hierarchy. LMAT’s fast run times allow the software to be run initially with default settings and quickly rerun on targeted subsets of reads as needed.

The reported LMAT tests focus on identifying known organisms in complex samples, which the results show still presents a major challenge. The use of relatively short reads (25–100 bases) indicate that even known sequences can be difficult to taxonomically classify when they represent short genetic elements conserved among multiple taxa. LMAT analysis on human clinical samples exhibits a high read label rate allowing the much smaller pool of unlabeled reads to be interrogated for more distant evolutionary relationships with other tools. For some environmental samples, more of the microbial contents are expected to be highly divergent from the reference database and populated with greater amounts of non-microbial eukaryotic DNA. In these cases lower, rates of read labeling are obtained. The Supplementary Material shows the high scoring read label rates (score ≥1) for four different environmental samples range from 10 to 60%, but relaxing the default minimum read label score threshold increases the read label rate to >50% in all cases. We find that the species identified by reads with low scores are frequently the same as those identified by high confidence, high scoring reads. One can pull out additional lower scoring reads for taxa that are likely present as indicated by the higher scoring reads, thus diminishing the fraction of unclassified reads. The flexibility of adjusting the score threshold without rerunning the whole analysis enables LMAT to rapidly screen large environmental datasets and obtain a large fraction of labeled reads.

Although the full library should be fast enough to run on large metagenomes, marker libraries still have a speed advantage. Recently published marker library approaches like MetaPhlAn rely on bacterial marker genes, which cannot be applied directly to other microbial contents. Other marker libraries like Sequedex (Berendzen *et al.*, 2012) rely on an individual marker sequence to contain the taxonomic signal in a single contiguous genetic element. By contrast, our method resolves the taxonomic signal across the entire read with a scoring procedure. Individual k-mers may contain part of the signal with the intersection of taxonomic labels from multiple k-mers yielding a stronger signal, and thus accuracy should improve with sequencer read length. The reference database size is a function of genetic and taxonomic diversity allowing near neighbors to be added with only a limited increase in the database size. As the number of neighbor strains increase, strain level discrimination run time costs grow. Although our method reports strain level discrimination, accuracy was measured at the species level because minimum genome coverage affects accuracy and the available synthetic test sets focus on species discrimination. Future work will include designing better strain discrimination tests and expand the database to include functional annotation.

## Supplementary Material

Supplementary Data
