# Supplementary material for: Scalable metagenomic taxonomy classification using a reference genome database
Source: Bioinformatics. 2013 Jul 4;29(18):2253–60. doi: 10.1093/bioinformatics/btt389 (PMC3753567; doi:10.1093/bioinformatics/btt389)
Supplement: Supplementary Data [file supp_29_18_2253__index.html]

Scalable metagenomic taxonomy classification using a reference genome database — Scalable metagenomic taxonomy classification using a reference genome database — Scalable metagenomic taxonomy classification using a reference genome database — Supplementary Data 

# Scalable metagenomic taxonomy classification using a reference genome database

## 

files

**Files in this Data Supplement:**

- Supplementary Data - pdf file
