# Supplementary material for: Scalable metagenomic taxonomy classification using a reference genome database
Source: Bioinformatics. 2013 Jul 4;29(18):2253–60. doi: 10.1093/bioinformatics/btt389 (PMC3753567; doi:10.1093/bioinformatics/btt389)
Supplement: Supplementary Data [file supp_btt389_supp1.pdf]

Scalable metagenomic taxonomy classification using a reference genome database: Supplementary Material

Sasha K. Ames, David A. Hysom, Shea N. Gardner, G. Scott Lloyd, Maya B. Gokhale, Jonathan E. Allen

1 Creation of the random model

N reads (N was set to 1,000,000) of length l were created by generating a sequence of A, C, G and T's assuming each base is equally likely. Each read is searched against the LMAT database and the classification table is created (see Figure 2 of the main text) to record the P_j proportion of k-mers in the read associated with each candidate taxonomic ID. Candidate IDs with a zero proportion are discarded, and no longer considered in subsequent random model creation. Each remaining taxonomic ID proportion is treated as a single observation. All of the randomly generated reads are examined and the highest proportion value P_j is chosen as the random value PR_j for taxonomy ID j. Since the GC content of a randomly generated read on average will be 50% and the GC content across the different taxon may differ considerably, a sufficiently large number of randomly generated reads is needed to observe a range of GC content values in the set of randomly generated reads. We chose a minimum threshold of 1000 reads with a non-zero proportion for each taxonomic ID to capture a range for possible GC content values.

Intuitively, taxonimc IDs associated with large numbers of k-mers such as NCBI taxonomy ID "131567" (cellular organisms) are expected to have many random reads with non-zero proportion, easily exceeding the threshold of 1000. In contrast, a highly strain specific taxonomic ID for a single small genome with relatively few distinct k-mers will be associated with very few random reads. The assumption is the maximum proportion of distinct k-mers is dominated by the number of k-mers available to match for the taxonomic ID. To get around this problem, multiple taxonomic IDs with similar numbers of distinct k-mers are grouped together. For example, taxonomic ID "40537" (Epsilon papillomavirus 1) is associated with one genome and only 8481 distinct 20-mers. The one million randomly generated reads will generate relatively few cases where a read's taxonomy IDs have a non-zero proportion value. Other taxonomic IDs with similar number of distinct k-mers will be grouped with "40537". As an example, taxonomic ID "335963" (Hippeastrum latent virus) is also reported to have 8481 distinct 20-kmers. The number of random reads with non-zero proportion for each taxonomic ID are checked and the maximum is taken from among the pooled group. In this example, if there were 500 random reads for "40537" and the maximum proportion value was 0.2 and there were 500 random reads for taxonomic ID "335963" and the maximum proportion value was 0.3, the two taxonomic IDs combined would form the minimum number of 1000 random read observations and both would be assigned the same random proportion value of 0.3 after taking the maximum between the two groups. In general, the program enumerates over the list of taxonomic IDs in increasing order sorted by the number of distinct k-mers and combines the taxonomic IDs into a group until the total number of random reads with non-zero proportion exceeds 1000.

It is important to consider the impact of nucleotide composition bias found in different genomes on the random model. A novel genome in the metagenomic query set with high GC content could share more k-mers with taxonomic IDs associated with high GC genomes. While generating 80 megabases of random reads (e.g. 1,000,000 reads of length 80) appeared to produce good results, there is a potential to under sample from high or low GC content random reads. To avoid the potential for under sampling the software supports drawing explicitly from a range of GC content values.

2 Impact of k on accuracy

Supplementary Figure 1 shows performance results for k considering a range from 17 to 20. Our initial hypothesis was that different values of k would perform differently for different test conditions. Results from five sample types are shown, the known bacteria composition case (PhymmBL data), novel viruses, novel prokaryotes, and novel protist and fungi (see main text for a description of the test data) Contrary to our initial expectation k=20 showed consistently better performance in terms of per read accuracy and false negative rate and motivated the choice of k=20 as the default. The lower false negative rate is explained by the ability to assign taxonomic labels with more significant (higher scoring) matches and more specific taxonomic labels, which outweighed the drawback of using higher values for k and higher numbers of reads which fail to match to the database due to the higher search seed size.

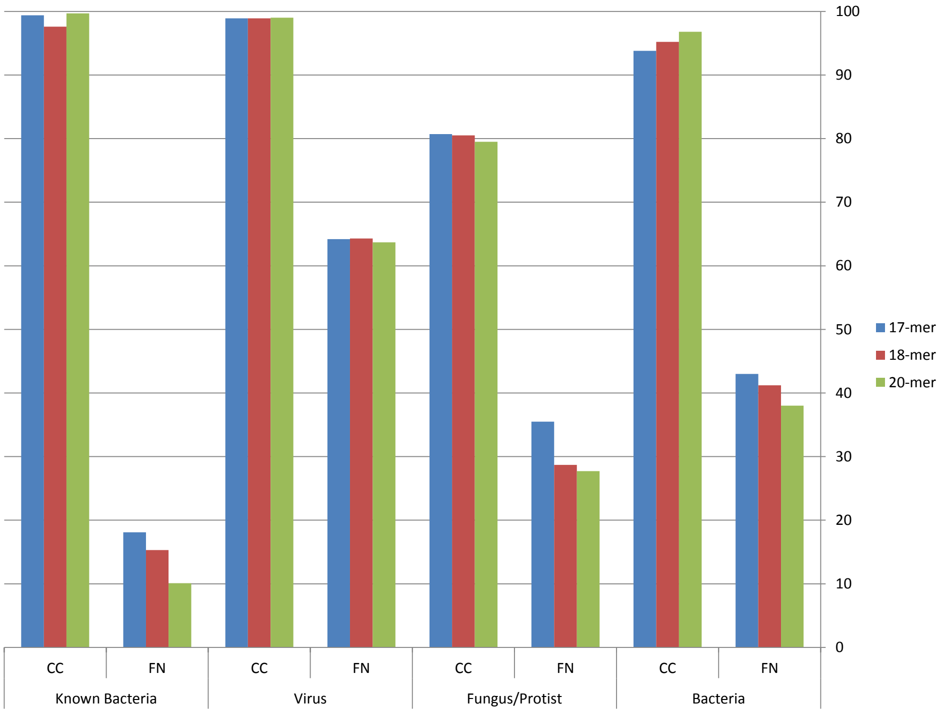

Supplementary Figure 1: Impact of k on accuracy. CC is the percentage of reads assigned a label that are correct. FN measures the percentage of reads that fail to be assigned a taxonomic label. Four test sets are shown, PhymmBL's published data set (Known Bacteria), the novel viral data set (Virus), eukaryotes with fungi and protist (Fungus/Protist) and the novel bacteria data set (Bacteria). Results using the same reference database (kFull) using three values of k (17,18 and 20) are shown.

Database	Species	Genus	Family	Order	Wrong	No Label	No Hits
Sample Type: Virus							
kFull	35.1 (99.7)	0.7 (97.9)	0.1 (100)	-	1.0	63.7	14.7
kML	22.7 (95.0)	-	-	-	5.8	75.9	54.6
Sample Type: Exclusive Novel Virus							
kFull	21.0 (98.0)	1.2 (97.7)	-	-	7.3	93.0	21.0
Sample Type: Eukaryotes							
kFull	35.6 (70.8)	9.6 (99.2)	0.5 (99.3)	4.3 (100)	20.5	13.0	3
kML	11.5 (52.8)	1.0 (96.4)	0.1 (95.0)	0.3 (98.8)	38.8	73	39.3
Sample Type: Prokaryotes							
kFull	43.0 (96.0)	8.0 (98.7)	1.7 (99.4)	0.3 (95.5)	3.2	38.0	1.3
kML	10.3 (85.2)	1.2 (99.6)	0.2 (99.1)	0.1 (94.2)	9.4	79.8	26.6
Sample Type: Exclusive Novel Prokaryotes							
kFull	30.6 (94.5)	4.1 (98.7)	2.8 (99.7)	0.3 (96.3)	4.3	54.2	2.2

Supplementary Table 1: Read accuracy for samples, which include novel species. Per rank accuracy shows two values - percentage of all reads correctly labeled by rank (Species, Genus, Family or Order) or incorrectly labeled (Wrong) or failed to be assigned a labeled (No Label and No Hits). In parentheses shows percentage of reads assigned a label at the specified rank that were correct. No Label = reads with no taxonomically informative label assigned, No Hits = reads with no k-mer matches to the database. - = entry not applicable. "Exclusive Novel" shows the subset of reads from non-exact match genomes shown in Supplementary Figure 2.

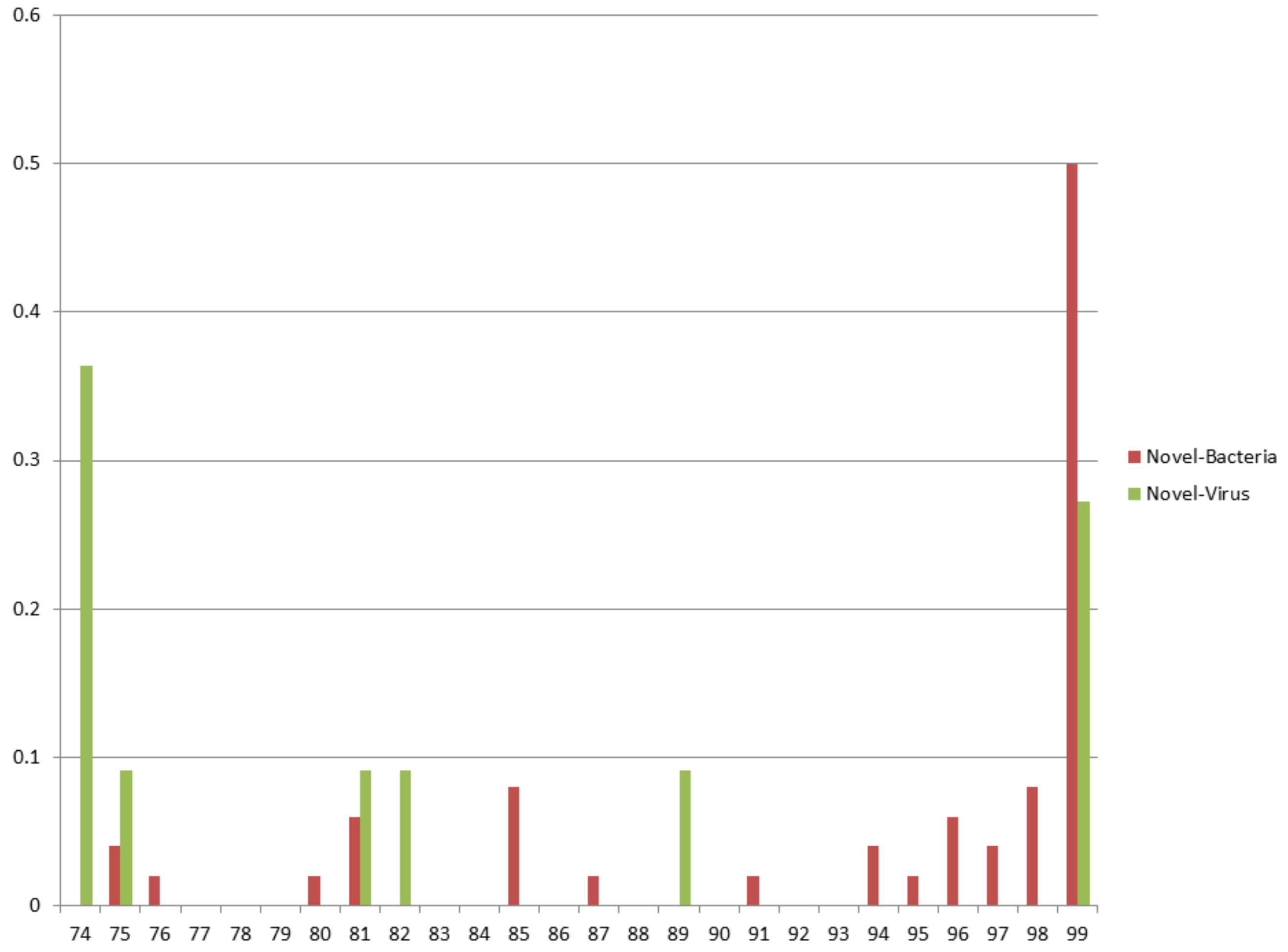

Supplementary Figure 2: Histogram of percent identity match between test organisms and their nearest match in the reference genome database. The fifty non-exact match genomes are shown.

3 Read taxonomy label accuracy for data sets with novel genomes

Supplementary Table 1 shows the individual read accuracy values, for the cases that include novel species. As expected, a higher percentage of reads were not assigned labels, however, for the cases where labels were assigned, accuracy rates were high. The one outlier was for the eukaryote case where 13% of reads (for the full library) belonging to *T. evansi* were assigned to *T. brucei*. The majority of the remaining wrongly labeled species with >100 labeled reads were distributed across other related eukayrotes: *Coccidioides posadasii*, *Trichophyton rubrum*, *Neosartorya fischeri*, *Entamoeba dispar*, and *Aspergillus flavus*. Supplementary Figure shows the Taxonomic distribution of the synthetic viral and bacterial datasets as Krona plots [Ondov *et al.* (2011)] and Supplementary Figure shows the histogram of the percent identity of to the nearest genome match in the LMAT reference database.

The query test genomes from the "Novel Bacteria" and "Novel Virus" were searched against the LMAT reference database using default blastn and the percent identity of the top hit was recorded. Fifty of the 100 test bacteria showed a 100% identity match. Eleven of the 25 virus genomes showed a 100% identity match with a genome in the reference database and 3 did not return a significant match. The percent identity of the non-exact match genomes relative to the closest match in the reference genome are shown as a histogram in Supplementary Figure 2. The genomes range in similarity from between 74% and 99% with genomes in the reference database. Supplementary Figure 3 and Supplementary Figure 4 show the taxonomic distribution of the different test genomes as a percentage of the number of reads taken from each genome. MetaSim was used to simulate equal concentrations of each test genome so microbes with larger genomes are represented with more reads than microbes with smaller genomes.

LMAT software was run on four environmental samples to consider how many reads would be assigned a taxonomic label in a non-human related metagenomic sample where the reference database may have poor genome representation for the relatively novel microbial environments. Datasets were downloaded from http://metagenomics.anl.gov/ [Meyer *et al.* (2008)] and are specified by identifiers: 68388 (rock), 37500 (soil-dessert), 37471 (soil-tropical) and 27017 (wastewater). Read lengths

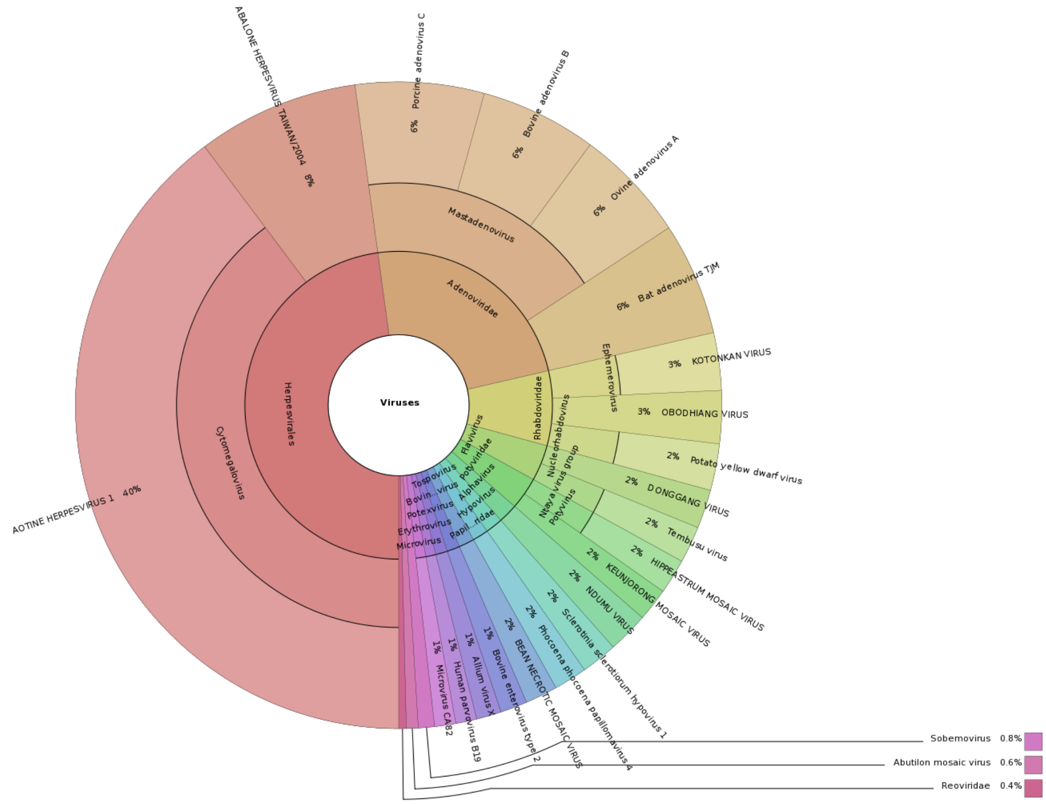

Supplementary Figure 3: Krona plot showing the taxonomic distribution of simulated reads in the "Novel Virus" test set.

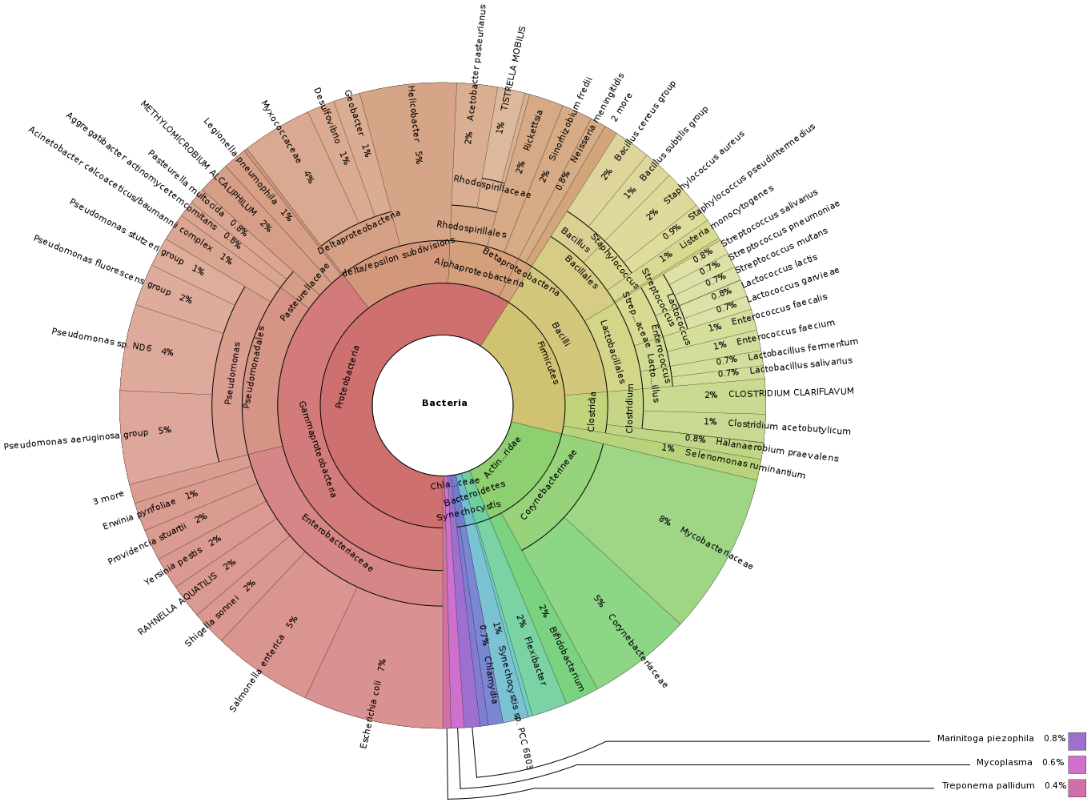

Supplementary Figure 4: Krona plot showing the taxonomic distribution of simulated reads in the "Novel Bacteria" test set.

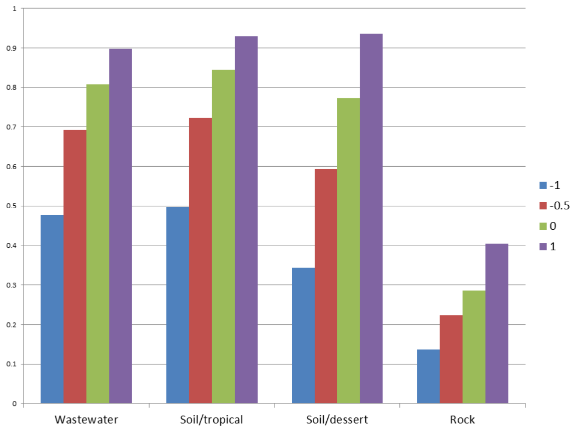

Supplementary Figure 5: Fraction of labeled reads that scored below the minimum threshold of -1,-0.5, 0 and 1. The y-axis shows the fraction of labeled reads for the four environmental datasets are examined. A higher fraction means more labeled reads were scored below the minimum threshold and are more divergent from the reference database.

were between 80 and 100 bases and number of reads per sample ranged from 55,500 to 6,535,257. Supplementary Figure 5 shows that the rock metagenomic sample, 60% of the reads were assigned a taxonomic label with a relatively high score of 1 or higher. For other environmental samples, lowering of the minimum score threshold is required in order to assign a taxonomic label to a majority of the reads in the sample. A future improvement to LMAT to recover more reads from novel genomes would be to automatically use information from the higher scoring taxonomic calls to determine which of the lower scoring reads are still good candidates for a taxonomic assignment. For example, the Iceman results illustrated how a read with a single 20-mer match to human was assigned a score of 0. It is difficult to completely rule out the possibility of a true match to another near neighbor genome not in the database (e.g. such as another primate genome). However, with the knowledge from other reads (and additional data) for the presence of human DNA, these relatively lower scoring reads can still in practice be assigned a taxonomic label.

4 Database Sizes

**Database**	kVB 18-mer	kVB 20-mer	kFull 17-mer	kFull 18-mer
Size	408	407	695	635
k-mer count	4.72	6.04	4.45	6.97
**Database**	kFull 20-mer	kML 17-mer	kML 18-mer	kML 20-mer
Size	619	93	39	4.5
k-mer count	9.21	1.26	0.633	0.078

Supplementary Table 2: Storage used (sizes in GB) and k-mer counts (billions) for various indexed database configurations. kVB databases contain virus and bacteria genomes only. kFull databases contain virus, bacteria, fungi and protist. kML are reduced size marker libraries derived from kFull.

Supplementary Table 2 shows the numbers of k-mers present and the total storage required for several of the databases we have created. The "Marker" databases have k-mers that have been specifically selected, while the other, larger databases contain every possible k-mer extracted from the original sequenced genomes in the reference database. The addition of eukaryotes (kFull vs kVB) increases the k-mer count and total size considerably. We also observe a slight reduction in size with the increase of k because despite an increase in k-mers, the average count of taxonomic information per k-mer decreases significantly. We are working on reducing the size of our indexed databases. The storage of taxonomic identifiers uses 6 bytes per taxon. We estimate this storage to use, for example 306 GB for the kFull 20-mer database. We have determined since the creation of these databases that the bytes per identifier can be reduced to 2. Thus, we approximate that the size of this database should be reduced to 413 GB.

Supplementary Table 3 shows the estimated increases in database size for the addition of several listed eukaryotic genomes. We present the number of k-mers and the estimated size for each genome. Note that size may appear smaller as we account for a percentage of k-mers shared with other genomes, which would not contribute to an increase in the hash table size, but does account for a small increase through several taxonomy identifiers stored per k-mer.

Genome	Total k-mers (millions)	est. additional size (GB)	cumulative size
Human	2,200	56	675
Chimpanzee	2,173	46	721
Turkey	892	23	744
Lizard	1,159	30	774
Fugu	313	8	782
Mosquito	216	6	788
Nematode	141	4	792
Rice	267	7	799

Supplementary Table 3: Estimated database size increase(s) from addition of multicellular Eukaryote genomes. Cumulative sizes add on to the kFull 20-mer database in Supplementary Table 2.

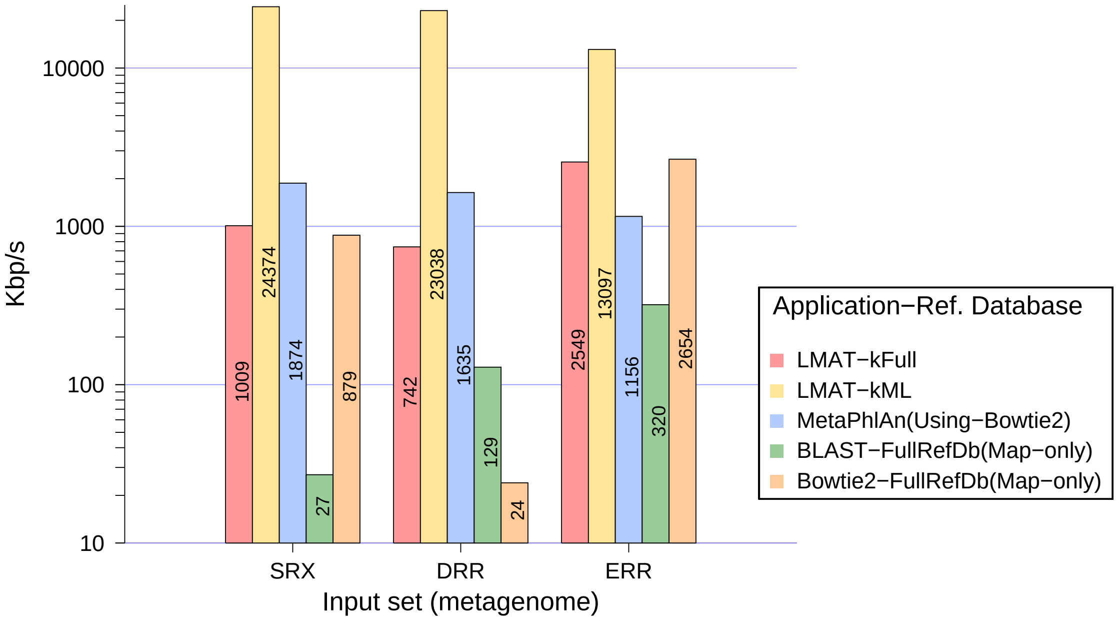

Supplementary Figure 6: Raw run time performance reported. Tests run on three real metagenomic data sets SRX, DRR and ERR. Run time is shown for three metagenomic classifiers (LMAT-kFull, LMAT-kML, MetaPhlAn using Bowtie2 and its database) and simple sequence searches for Bowtie and blastn (BLAST). Note log scale on y-axis; values given within each bar.

5 Run time Performance

Supplementary Figure 6 shows the raw performance of LMAT compared with the three search tools. Supplementary Table 4 shows the percentage of reads labeled (our method and MetaPhlAn) plus the percentage of raw matches from Bowtie searched against the same reference database as our full database classifier. Raw Bowtie searches against ERR011121 showed a much lower number of matched reads, which explain its faster over all performance in the test. Supplementary Table 5 shows the rank specificity of the labeled reads for ERR, which shows that 65.3% of the reads are assigned a species label.

6 Tyrolean Iceman Metagenome Analysis

All reads were downloaded from the NCBI Short Read Archive using accession identifiers ERR069107, ERR069108, ERR069109, ERR107307, ERR107308 and ERR107309. LMAT was initially run on all 2.3 billion reads, however, due to an observed large number of lower quality sequencer derived quality scores ($Q < 20$), results are reported on a filtered data set of 1.7 billion reads. The 2.3 billion reads were quality trimmed with FASTX Toolkit to remove reads with more than 60 percent of the bases having quality scores less than 20. The software program seqtk was then used to mask (replace base call with 'N') all base calls with quality value less than 20. Results running LMAT with a minimum read label score threshold of 0 and other default parameter settings (comparable score cutoff of one standard deviation and maximum of 50 candidate taxonomy identifiers per k-mer) are shown in Supplementary Figures 7, 8, and 9. Results are reported graphically using Krona

Input metagenome:		SRX022172	DRR00184	ERR011121
Read length:		100	75	50
Sample size (reads):		4,216,970	7,631,281	31,564,747
**Application**	**Database**	% labeled	% labeled	% labeled
LMAT	kFull	99.1%	99.4%	85.6%
LMAT	kML	47.6%	19.2%	58.0%
bowtie2	kFull	93.1%	98.6%	34.3%
bowtie2	MetaPhlAn	1.4%	1.23%	0.89%
blastn	kFull	79.6%	93.9%	20.8%

Supplementary Table 4: Percentages of reads matched (bowtie, blastn) or labeled (LMAT) measured for several databases and inputs.

Database	Species	Genus	Family	Order	No Label
kFull	65.3	8.5	1.2	3.2	14.4

Supplementary Table 5: Rank specificity for the ERR011121 dataset (listed in Supplementary Table 4). Shows the percentage of all reads labeled by rank (Species, Genus, Family or Order) or failed to be assigned a labeled (No Label).

[Ondov *et al.* (2011)]. Non NCBI taxonomy classification labels reported in the figures are “No Match”, which indicate that a read’s constituent k-mers were not found in the database, “Low Score” where the best matched score remains below a user defined significance threshold (defaults to 1) and “Root”, which means the read is matched with significant hits across multiple kingdoms. Note the relatively high number of NoMatch reads (45%) in Supplementary Figure 7 are not due to the lack of representation in reference genome database but rather are indicative of the high numbers of short reads interspersed with N’s to mask low quality base calls, which can remove all valid candidate k-mers from a read. The read label score is calculated by selecting null-models determined by the number of valid k-mers in the read so that long reads with relatively few valid k-mers do not receive an artificially contrived low score.

Supplementary Figure 10 shows the distance based neighbor joining tree for the Borrelia reads and all available Borrelia genomes. The phylogenetic tree corresponds well with LMAT’s classification in Supplementary Figure 9, showing that the majority of the reads do not appear to be uniquely associated with *B. burgdorferi*. The SNP tree identifies homologous genomic regions with a minimum of 8 bases on either side of the SNP using previously published methods [Gardner and Slezak (2010)]. Note that *B. crocidurae* is identified in the SNP tree but not in the LMAT classification since this genome was made available after the creation of the LMAT reference database. The second more current reference database was used as a post validation step to ensure that the Borrelia classified reads would not be uniquely matched to a newly sequenced genome. Reads were mapped to all available Borrelia genomes using BLAST (and -task blastn-short default parameter settings). Coverage of the respective reference genomes from the BLAST matched reads did not indicate a preference for one species over another.

7 Impact of sequencing error on taxonomy classification

While a single sequencing error alters up to 20 k-mers (when k=20), since the k-mers are bit-encoded in a 64 bit word, a single error generates 20 very different encodings, which are unlikely to be associated with a single specific taxonomic identifier. The Iceman data set, which is an extreme case of short reads with high error rates shows that error prone reads are classified but a higher percentage of reads go unclassified due to the cases where a short read has very few valid k-mers. It is possible for a sequencing error to lead to the misclassification of an individual read and this could explain some errors reported in the read-level accuracy rates.

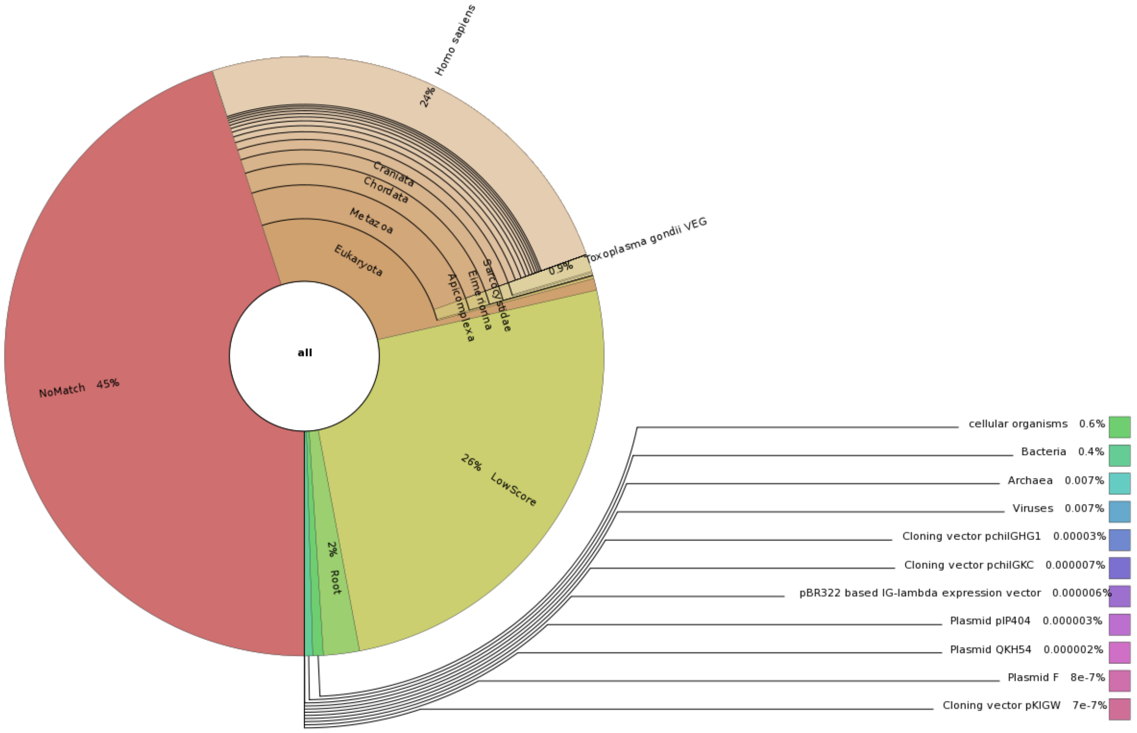

Supplementary Figure 7: Label distribution for all 1.729040669 billion reads using a minimum read label score of 0. The large number of "NoMatch" labeled reads indicate short reads with masked bases leading to reads with few or no valid 20-mers available to search against the database. A large portion (26%) of the reads were assigned a low read label score also due to the relatively low number of valid 20-mers present in many short reads. Results show 0.5% of the reads are uniquely associated with Bacteria. *Toxoplasma gondii* reads were determined to be attributed to human contamination in reference draft genomes. Thus, no non-human eukaryote calls were made.

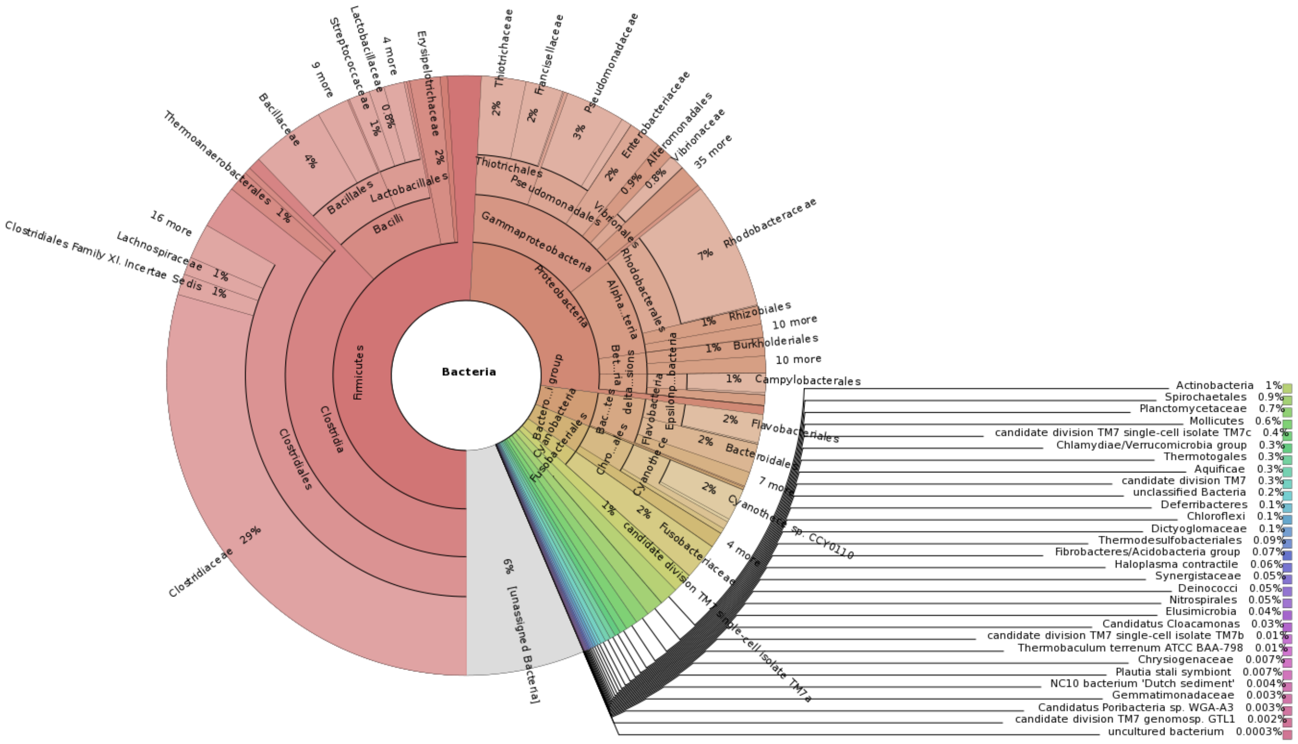

Supplementary Figure 8: Label distribution for 7,496,855 reads labeled as Bacteria with a read label score greater than 0.

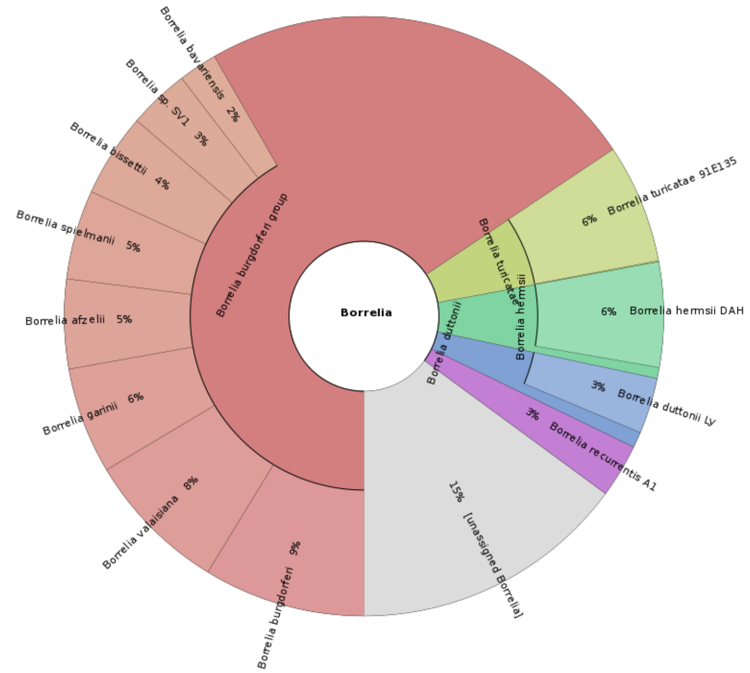

Supplementary Figure 9: Label distribution for the 16,180 Borrelia genus specific reads labeled with read label score greater than 0.

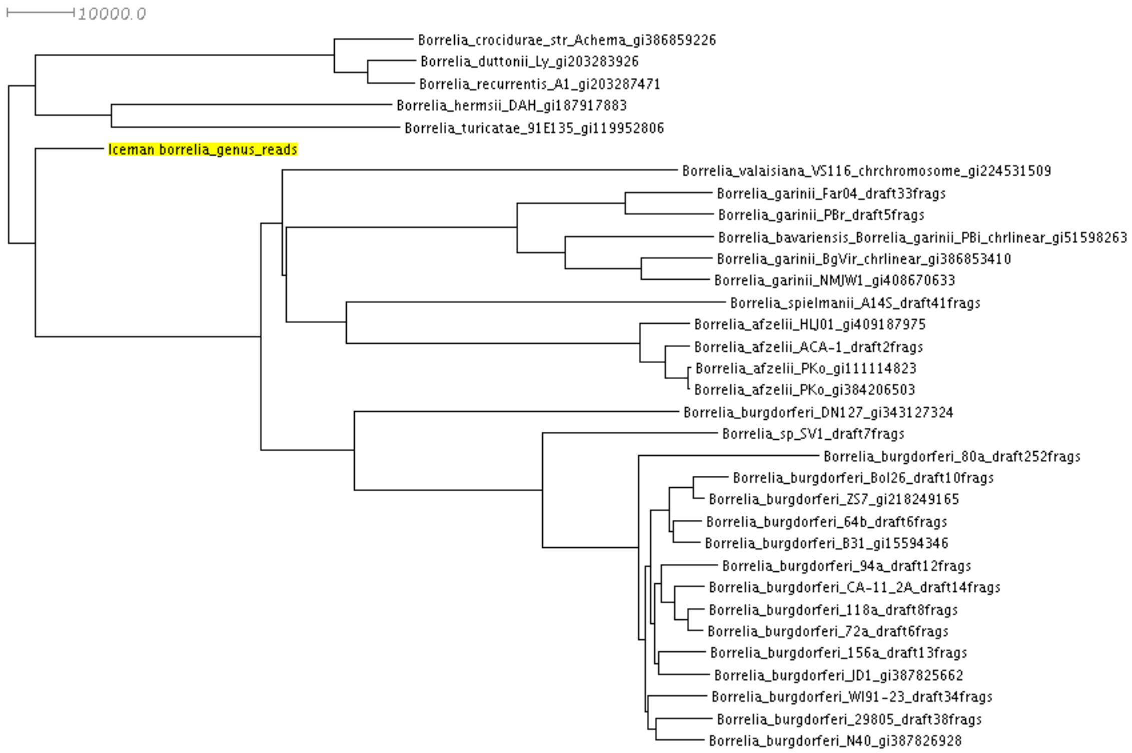

Supplementary Figure 10: SNP tree comparing Borrelia genus specific reads with comparable regions from all other sequenced Borrelia genomes.

8 Additional information on methods testing

The previously unpublished data sets used for testing against novel species (Virus, Bacteria, and Eukaryotes) are made publicly available along with a list of all of the GenBank identifiers found in our reference genome database. For convenience, we also make an 18-mer version of the marker library in memory mapped form available for immediate use. The data can be retrieved from ftp://gdo-bioinformatics.ucllnl.org/lmat

Third party software versions used in testing:

- PhymmBL - 3.2
- MetaPhlAn - 1.6.0
- Genometa - 0.51
- Bowtie2 - 2.0.0-beta6
- NCBI BLAST - 2.2.27+ (unless otherwise specified default settings were used)

References

[Ondov *et al.* (2011)] Ondov, B., *et al.* (2011) Interactive metagenomic visualization in a web browser. BMC Bioinformatics, 12(1), 385.

[Gardner and Slezak (2010)] Gardner S.N. and Slezak T. (2010) Scalable SNP analyses of 100+ bacterial or viral genomes. J Forensic Research, 1(107).

[Meyer *et al.* (2008)] Meyer, F., *et al.* (2008) The metagenomics RAST server - a public resource for the automatic phylogenetic and functional analysis of metagenomes. BMC Bioinformatics, 9:386.
